# ¿Son los anticuerpos IgG e IgM contra los antígenos S y N del SARS-CoV-2 siempre predictores de infección previa por SARS-CoV-2?

**DOI:** 10.1515/almed-2023-0036

**Published:** 2023-05-05

**Authors:** Giuseppe Lippi, Brandon M. Henry, Laura Pighi, Simone De Nitto, Gian Luca Salvagno

**Affiliations:** Departamento de Bioquímica Clínica, Universidad de Verona, Verona, Italia; Laboratorio Clínico, Sección de Nefrología e Hipertensión, Cincinnati Children’s Hospital Medical Center, Cincinnati, OH, USA; Servicio de Medicina de Laboratorio, Pederzoli Hospital, Peschiera del Garda, Italia

**Keywords:** anticuerpos, COVID-19, infección, SARS-CoV-2, serología

## Abstract

**Objetivos:**

Evaluamos si los inmunoensayos con anticuerpos IgG e IgM contra las proteínas *spike* (S) y nucleocápside (N) del SARS-CoV-2 detectan infecciones previas por SARS-CoV-2.

**Métodos:**

Analizamos una cohorte de profesionales sanitarios que había completado el ciclo de vacunación. Desde 2020, y cada 2–4 semanas, se les realizaron revisiones médicas y pruebas moleculares para diagnosticar una posible infección por SARS-CoV-2. Se extrajeron muestras de sangre venosa para medir los niveles de anticuerpos contra el SARS-CoV-2 con los ensayos MAGLUMI^®^ 2019-nCoV lgG y 2019-nCoV lgM CLIA dirigidos a las proteínas S y N del SARS-CoV-2.

**Resultados:**

En total, la prueba RT-PCR fue positiva para SARS-CoV-2 en 31 (58,5 %) sujetos (el resultado fue positivo una vez en 24 sujetos y dos veces en 7). No se observó una relación directa entre los niveles de anticuerpos IgM contra S y N del SARS-CoV-2 y la positividad de la prueba molecular. El análisis de regresión univariante reveló una relación estadísticamente significativa entre los anticuerpos IgG contra S y N del SARS-CoV-2 y una prueba molecular positiva (r=0,33; p=0,015) y el número de pruebas moleculares positivas (r=0,43; p=0,001). Sin embargo, no se observó correlación con el número de dosis de la vacuna (r=−0,12; p=0,392). La significación se mantuvo en el análisis de regresión lineal (p=0,029 y p<0,001, respectivamente) tras controlar el efecto del sexo, edad, índice de masa corporal y dosis de la vacuna. En el análisis de la curva ROC, los IgG contra S y N del SARS-CoV-2 predijeron significativamente la positividad de la prueba molecular (AUC, 0,69; IC95 %; 0,55–0,84). El mejor valor umbral fue 0,05 AU/mL, con una precisión del 67,9 %, una sensibilidad del 0,97, y una especifidad de 0,27.

**Conclusiones:**

Aunque los anticuerpos IgG contra S y N del SARS-CoV-2 proporcionan información útil para identificar infecciones previas por SARS-CoV-2, se debería emplear un valor umbral inferior al de la reactividad de la muestra. Los anticuerpos IgM contra S y N del SARS-CoV-2 no son válidos para tal fin.

## Introducción

La cuantificación de anticuerpos contra el SARS-CoV-2 (coronavirus de tipo 2 causante del síndrome respiratorio agudo severo) se utiliza frecuentemente en lugar de la prueba molecular para identificar infecciones previas por SARS-CoV-2 [1]. Para detectar esta infección cuando se han administrado vacunas que no contenían la secuencia (o parte de la secuencia) de la proteína N, en lugar de determinar los anticuerpos contra la proteína *spike* (S) del virus, se han cuantificado los anticuerpos contra la proteína nucleocápside (N) del SARS-CoV-2. De este modo, la identificación de anticuerpos contra la proteína N del SARS-CoV-2 permite detectar la presencia de una respuesta inmune contra el virus y, teóricamente, clasificar a los sujetos como “infectados” o “infectados previamente” por SARS-CoV-2 [2], [3], [4].

Sin embargo, diversos aspectos (esto es, el desarrollo y detección inconstante en sujetos infectados por nuevas cepas de SARS-CoV-2 con muchas mutaciones, la disminución de los niveles séricos, la falta de umbrales de “positividad” validados, etc.) cuestionan si los niveles de anticuerpos contra el SARS-CoV-2 son un marcador fiable de infección natural previa en sujetos que no han recibido una vacuna que contenga la secuencia de la proteína N, especialmente cuando dichos resultados se utilizan para orientar las políticas de salud pública [5]. Se realizó un estudio para comprobar si los inmunoensayos de cuantificación de niveles de anticuerpos IgG e IgM contra las proteínas *spike* (S) y nucleocápside (N) del SARS-CoV-2 detectan infecciones previas por SARS-CoV-2.

## Materiales y métodos

La población de estudio estaba compuesta por una cohorte de profesionales sanitarios del Hospital Pederzoli (Peschiera del Garda, Verona, Italia), que habían recibido un ciclo de vacunación completo con la vacuna de ARNm bivalente BNT162b2 de Pfizer/Biontech, entre noviembre y diciembre de 2022. Desde 2020, y cada 2–4 semanas, se les realizaron pruebas moleculares a todos los sujetos (Allplex SARS-CoV-2 de Seegene; kit de RT-PCR para SARS-CoV-2 de Seegene Inc., Corea del Sur, o Altona Diagnostics RealStar, Altona Diagnostics GmbH, Hamburgo, Alemania) para detectar infecciones por SARS-CoV-2, con los métodos descritos anteriormente [6]. Antes de administrar la vacuna bivalente contra el COVID-19, se extrajo sangre venosa para cuantificar los anticuerpos contra el SARS-CoV-2 con la prueba MAGLUMI 2019-nCoV lgG/IgM CLIA (SNIBE; Shenzhen, China). Según el fabricante, la prueba detecta los anticuerpos IgM o IgG contra las proteínas S y N del SARS-CoV-2 (esto es, S/N). Un resultado ≥1,1 (absorbancia de la muestra/absorbancia del calibrador) se considera reactivo. Según evaluaciones previas de estos inmunoensayos, la repetibilidad es <6 %, la imprecisión intermedia es <6 %, mientras que la sensibilidad a la hora de detectar infecciones agudas por SARS-CoV-2 es de 1,00 para los IgG y 0,88 para los IgM [7]. Existen más datos e información técnica sobre estos inmunoensayos en la literatura [7, 8].

Todos los resultados se expresan como mediana y rango intercuartílico (RIC). El análisis estadístico se realizó con Analyse-it (Analyse-it Software Ltd, Leeds, Reino Unido), con análisis univariante (correlación de Spearman, prueba U de Mann-Whitney) y multivariante (regresión lineal múltiple). Todos los sujetos de este estudio observacional prospectivo firmaron un consentimiento informado. El estudio se realizó con ajuste a los principios de la Declaración de Helsinki. El protocolo fue aprobado por el Comité Ético de las Provincias de Verona y Rovigo (59COVIDCESC; 8 de noviembre de 2021).

## Resultados

**Figura 1:**
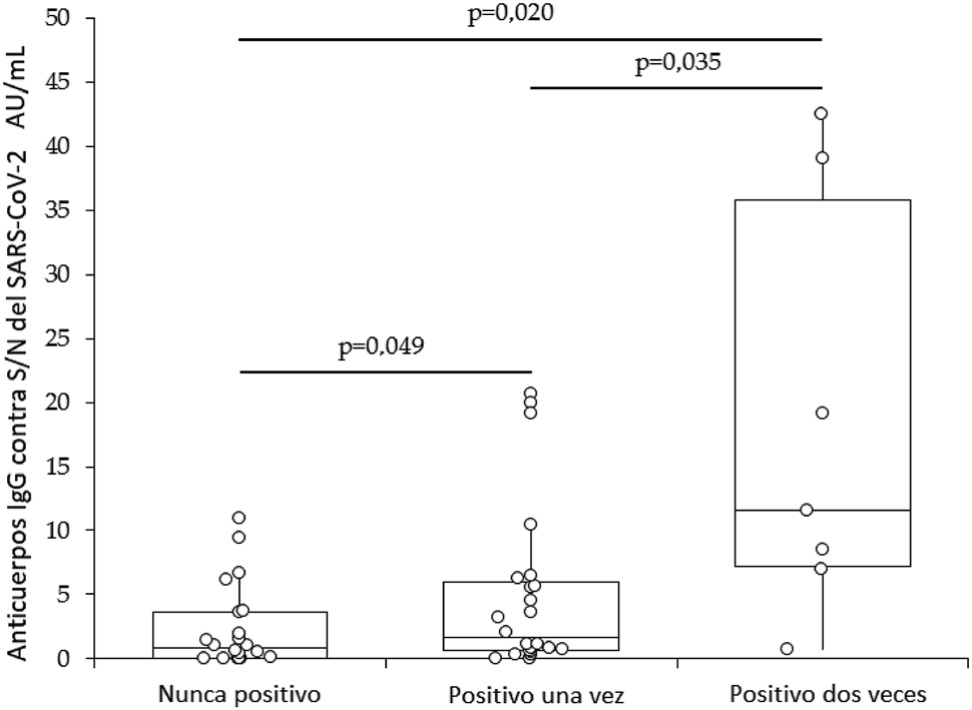
Anticuerpos IgG contra S/N del SARS-Cov-2 en sujetos sin resultado molecular positivo, con un resultado positivo, y con dos resultados para infección por SARS-CoV-2. SARS-CoV-2, coronavirus de tipo 2 causante del síndrome respiratorio agudo severo.

La población final de estudio estaba compuesta por 53 sujetos (mediana de edad, 43 años; RIC, 33–56 años; 27 mujeres) (Tabla 1). La prueba RT-PCR fue positiva para SARS-CoV-2 en 31 (58,5 %) sujetos (el resultado fue positivo una vez en 24 sujetos y dos veces en 7). El análisis univariante no reveló ninguna correlación entre los niveles de anticuerpos IgM contra S y N del SARS-CoV-2 y el resultado de la prueba molecular (r=−0,25; entre −0,48 y 0,03; p=0,074). No se observaron diferencias significativas en la mediana de anticuerpos IgM contra S y N entre sujetos con (0,09; IC95 %, 0,01–0,15) o sin (0,17; IC95 %, 0,09–0,23) resultado positivo en la prueba molecular (p=0,076). El análisis de regresión univariante reveló una relación estadísticamente significativa entre los anticuerpos IgG contra S y N y una prueba molecular positiva (r=0,33; IC95 %, 0,07–0,55; p=0,015) y el número de pruebas moleculares positivas (r=0,43; IC95 %, 0,18–0,63; p=0,001). Sin embargo, no se observó correlación con el número de dosis de la vacuna (r=−0,12; IC95 %, −0,38–0,16; p=0,392). Ni la edad (0.21; IC95 %, −0,06–0,46; p=0,122), ni el sexo (r=−0,10; IC95 %, −0,36–0,18; p=0,476), ni el índice de masa muscular (BMI; r=0,19; IQR, −0,08 a 0,44; p=0,163) mostraron correlación con los anticuerpos IgG contra S y N. La asociación entre los anticuerpos IgG contra S y N y la positividad de la prueba molecular y el número de pruebas moleculares positivas continuó siendo significativa en el análisis de regresión lineal múltiple (p=0,029 y p<0,001, respectivamente), tras controlar el efecto del sexo, edad, índice de masa corporal y dosis de la vacuna. La mediana de anticuerpos IgG contra S y N del SARS-CoV-2 aumentó progresivamente en sujetos con una prueba molecular negativa (0,84 AU/mL; RIC, 0,04–3,62 AU/mL), una prueba molecular positiva (1,61 AU/mL; RIC, 0,61–5,99 AU/mL), y dos pruebas moleculares positivas (11,54; AU/mL; RIC, 7,20–35,77). La relación se mantuvo estadísticamente significativa en todo momento entre todos los grupos (Figura 1).

**Tabla 1:** Características principales de la población de estudio. Los valores están expresados en medianas y rango intercuartílico (RIC).

Parámetros	Valor
n	51
Edad, años	43 (33–56)
Mujeres	27 (50,9 %)
IMC, kg/m^2^	24,7 (23,0–26,2)
Dosis de la vacuna	
– Una	4 (7,5 %)
– Dos	0 (0 %)
– Tres	49 (92,5 %)
RT-PCR positiva para infección previa por SARS-CoV-2	31 (58,5 %)
– Positiva una vez	24 (45,3 %)
– Positiva dos veces	7 (13,2 %)
IgM contra S/N del SARS-CoV-2, AU/mL	0,11 (0,03–0,21)
IgG contra S/N del SARS-CoV-2, AU/mL	1,54 (0,46–6,42)

IMC, índice de masa corporal; SARS-CoV-2, SARS-CoV-2, coronavirus de tipo 2 causante del síndrome respiratorio agudo severo.

**Figura 2:**
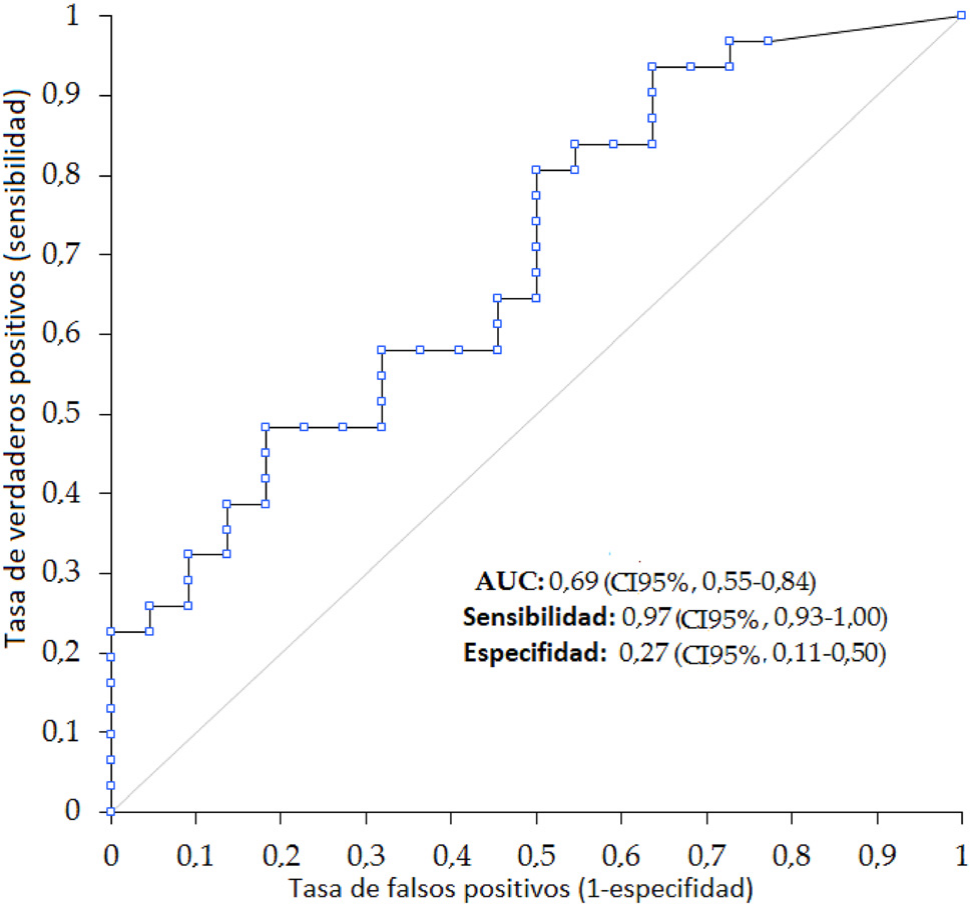
Análisis de curva ROC para niveles de anticuerpos IgG contra el antígeno S/N del SARS-Cov-2 para identificar positividad de la prueba molecular para infección previa por SARS-CoV-2. SARS-CoV-2, coronavirus de tipo 2 causante del síndrome respiratorio agudo severo.

En el análisis de la curva ROC, los IgG contra S y N predijeron significativamente la positividad de la prueba molecular (área bajo la curva [AUC], 0,69; IC95 %; 0,55–0,84). El mejor valor umbral, 0,05 AU/mL, mostró una precisión del 67,9 % (IC95 %, 53,7–80,1 %), una sensibilidad del 0,97 (IC95 %, 0,93–1,00), y una especifidad de 0,27 (IC95 %, 0,11–0,50) (Figura 2). La precisión, sensibilidad y especifidad con el valor umbral de reactividad de los anticuerpos IgG contra S y N (≥1,1 AU/mL) fueron del 60,4 % (IC95 %, 46,0–73,6 %); 0,65 (IC95 %, 0,45–0,81); y 0,55 (IC95 %, 0,32–0,76), respectivamente. Se podría alcanzar una especifidad muy alta para una prueba molecular positiva incrementando el valor umbral a 6,93 AU/mL, logrando así una especifidad de 0,91 (IC95 %, 0,17–0,51); una sensibilidad de 0,32 (IC95 %, 0,17–0,51); y una precisión general de 56,6 % (IC95 %, 42,3–70,2 %) (Tabla 2).

**Tabla 2:** Precisión diagnóstica, sensibilidad y especifidad de los valores umbral de anticuerpos IgG contra S/N del SARS-CoV-2 S/N para identificar a sujetos con una prueba molecular positiva.

Valor umbral	Precisión	Sensibilidad	Especifidad
0,05 AU/mL	67,9 % (CI95 %, 53,7–80,1 %)	0,97 (CI95 %, 0,93–1,00)	0,27 (CI95 %, 0,11–0,50)
1,10 AU/mL	60,4 % (CI95 %, 46,0–73,6 %)	0,65 (CI95 %, 0,45–0,81)	0,55 (CI95 %, 0,32–0,76)
6,93 AU/mL	56,6 % (CI95 %, 42,3–70,2 %)	0,32 (CI95 %, 0,17–0,51)	0,91 (CI95 %, 0,17–0,51)

SARS-CoV-2, coronavirus de tipo 2 causante del síndrome respiratorio agudo severo.

## Discusión

La generación de anticuerpos específicos contra la proteína N (nucleocápside), especialmente los IgG, es característica de los pacientes convalecientes de una infección aguda por SARS-CoV-2. El análisis publicado por el Grupo Cochrane de Trabajo sobre Precisión de las Pruebas Diagnósticas de COVID-19 señala que el 90 % de los sujetos convalecientes de una infección aguda generan anticuerpos IgG contra el SARS-CoV-2, mientras que un porcentaje mucho menor (alrededor del 70 %) desarrolla anticuerpos IgM [4]. De este modo, los IgG contra el SARS-CoV-2 se postulan como una medida más sólida y fiable de evaluar la inmunidad adquirida contra el SARS-CoV-2 que los anticuerpos IgM contra el antígeno N del SARS-CoV-2. Esto también se refleja en el hecho de que los anticuerpos IgM contra N disminuyen a una velocidad considerablemente mayor que los IgG, tal como demuestran estudios anteriores [9, 10]. Así, la producción de anticuerpos IgM contra N del SARS-CoV-2 es mucho menos frecuente que la de anticuerpos IgG contra N. Además, los IgM tienden a disminuir con mayor rapidez, lo que los convierte a los primeros en un marcador poco fiable de infección previa por SARS-CoV-2. Por otro lado, la respuesta de los IgG contra N del SARS-CoV-2 parece ser más frecuente y estable. Pushpakumara y col. observaron que los anticuerpos IgG contra N son detectables en la gran mayoría de los sujetos con infección natural previa o vacunados con vacunas de virus inactivado [11]. Dhakal y col evaluaron a 182 adultos (101 vacunados y posteriormente infectados, 28 infectados y posteriormente vacunados, y 53 no vacunados en ningún momento) [12], y observaron que los pacientes infectados por SARS-CoV-2 previamente a la vacunación o que jamás se habían vacunado presentaban niveles persistentemente elevados de anticuerpos IgG contra N del SARS-Cov-2, mientras que los que habían recibido la vacuna antes de contraer la infección mostraban valores significativamente menores de estos anticuerpos (p<0,001 en cada caso). Sin embargo, en otro estudio prospectivo se observó que los niveles de anticuerpos IgG contra N del SARS-Cov-2 parecían disminuir antes que los niveles de anticuerpos IgG contra S, especialmente en los pacientes mayores [13], lo que cuestiona si esta prueba tiene suficiente sensibilidad para identificar a los sujetos con infección previa por SARS-CoV-2.

Los resultados del presente estudio aportan respuestas relevantes a dichas cuestiones. En primer lugar, no hallamos una asociación significativa entre los niveles de anticuerpos IgM contra los antígenos S y N del SARS-CoV-2 y la positividad de la prueba molecular (también se observó que sus medianas eran paradójicamente más bajas en aquellos sujetos con una prueba molecular positiva que en los que obtuvieron un resultado negativo), lo que nos permite rápidamente excluir esta prueba de análisis posteriores, debido a su muy poco fiable significación clínica. Así mismo, demostramos que el valor umbral de reactividad de los anticuerpos IgG contra los antígenos S y N del SARS-CoV-2 (esto es, 1,1 AU/mL), establecido específicamente para detectar la respuesta humoral inmune durante o inmediatamente después de una infección aguda, no demostró tener un rendimiento diagnóstico adecuado a la hora de detectar la positividad de una prueba molecular en un periodo cercano a los tres años. Concretamente, la precisión general y sensibilidad para identificar infección previa (esto es, con una prueba molecular positiva) fueron del 60,4 % y el 0,65, mientras que un valor umbral mucho más bajo (esto es, 0,05 AU/mL) permitió incrementar la precisión y sensibilidad al 67,9 % y a 0,97, respectivamente. No obstante, esta mayor sensibilidad se logró a expensas de una menor especifidad, que disminuyó de 0,55 a 0,27. Esto significa que el valor umbral de 0,05 AU/mL permitiría identificar prácticamente a la totalidad de los sujetos con infección previa por SARS-CoV-2, a expensas de obtener un porcentaje considerablemente alto de falsos positivos (alrededor del 35 %). El uso de un valor umbral más alto (6,93 AU/mL) permitiría descartar infecciones previas (esto es, una prueba molecular positiva) con una especifidad muy alta (superior a 0,90), aunque se obtendrían multitud de falsos negativos (51 %). Schaffner y col analizaron la trayectoria de los anticuerpos IgG contra el antígeno N del SARS-CoV-2 en 82 sujetos con infección por SARS-CoV-2 confirmada en el laboratorio y observaron que, utilizando el índice umbral sugerido por el fabricante, la sensibilidad disminuía a 0,91 a los 48 días, y a 0,63 a los 140 días [14]. Esto refuerza los resultados de otros estudios, como el publicado por Krutikov y col [15], que identificaron una mediana de tiempo de unos 242 días para la reversión de los niveles de anticuerpos IgG contra los antígenos N del SARS-CoV-2. Esto significa que, al año de haber desarrollado infección aguda, estos anticuerpos disminuyen por debajo de los valores de reactividad en casi la mitad de los sujetos. El estudio longitudinal de Loesche y col. mostró una serorreversión sustancial de los anticuerpos totales contra el antígeno N del SARS-CoV-2 a los 18 meses de la infección [16].

Transcurridos más de tres años desde el inicio de la pandemia, sigue sin haberse establecido una estrategia diagnóstica clara para el COVID-19. Aunque se sabe que los anticuerpos contra el SARS-CoV-2 tienen una significación clínica modesta en el diagnóstico de infección aguda, siguen siendo útiles para el seguimiento de la respuesta humoral [17]. Sin embargo, cuando se utilizan en los estudios serológicos, se deberían establecer valores umbral “móviles” de IgG contra el antígeno N del SARS-CoV-2, en función del método y propósito de la prueba. Así, se deberían emplear valores más bajos para identificar a los sujetos con infección previa por SARS-CoV-2, y valores más altos para identificar a sujetos que no han pasado la infección por SARS-CoV-2.

En conclusión, aunque los anticuerpos IgG contra los antígenos S y N del SARS-CoV-2 pueden ayudar a detectar infecciones por SARS-CoV-2, se debería emplear un valor umbral más bajo que el de reactividad de la muestra, tal como sugieren estudios anteriores [18]. Los anticuerpos IgM contra los antígenos S y N del SARS-CoV-2 no resultan útiles para este propósito.
